# Multiple drug resistance bacterial isolates and associated factors among urinary stone patients at the University of Gondar Comprehensive Specialized Hospital, Northwest Ethiopia

**DOI:** 10.1186/s12894-021-00794-8

**Published:** 2021-02-23

**Authors:** Desie Kasew, Setegn Eshetie, Abeje Diress, Zerubabiel Tegegne, Feleke Moges

**Affiliations:** 1 Department of Medical Microbiology, School of Biomedical and Laboratory Sciences, College of Medicine and Health Sciences, University of Gondar, P.O. Box 196, Gondar, Ethiopia; 2 Department of Radiology, School of Medicine, College of Medicine and Health Sciences, University of Gondar, P.O. Box 196, Gondar, Ethiopia

**Keywords:** Urinary stone, Multiple drug resistance, Urinary tract infection

## Abstract

**Background:**

The urinary stone and urinary tract infection (UTI) are invariably associated and are frequent causes of morbidity. Date on burden of UTI among urinary stone patients is lacking in Ethiopia. This study was aimed to assess bacterial profile, antimicrobial susceptibility and associated factors among urinary stone patients at the University of Gondar Comprehensive Specialized Hospital.

**Methods:**

An institution based cross sectional study was conducted. Basic sociodemographic data were collected using a structured questionnaire. Bacterial identification of uropathogens and drug susceptibility testing were done following standard microbiological techniques. The data were entered and analyzed using SPSS version-23. Bivariate and multivariate logistic regressions were used to identify possible associated risk factors. Results with *P* value < 0.05 was considered statistically significant.

**Result:**

A total of 300 urinary stone patients were enrolled. Of these, 153 (51%) were male and 261(87%) were urban residents. The overall prevalence of urinary tract infection was 49 (16.3%) (95% CI 12–21%). A high level of resistance was observed to ampicillin, penicillin and trimethoprim-sulfamethoxazole while majority of isolates were most sensitive to nitrofurantoin and ciprofloxacin. Multi-drug resistant isolates were 16/49 (32.7%), 75% of them being Enterobacteriaceae isolates. More than one-third 9/26 (34.6%) of Gram-negative isolates were Extended Spectrum Beta-Lactamase (ESBL) producing *E. coli* and *K. pneumoniae*. Being female, history of urinary tract infection and history of drug use were the independent risk factors.

**Conclusion:**

Most of the bacterial isolates from urinary stone patients were resistant to ampicillin, penicillin and trimethoprim-sulfamethoxazole. *E. coli* and *K. pneumoniae* were the most common extended spectrum beta-lactamase producing isolates. Sex, history of urinary tract infection and previous drug use were found to be risk factors. Routine diagnosis of urinary stone patients for urinary tract infection should be promoted and further researches are encouraged.

**Supplementary Information:**

The online version contains supplementary material available at 10.1186/s12894-021-00794-8.

## Background

Urinary stone and urinary tract infections (UTI) are constantly associated complaints of the urinary system [1]. Urinary stones also known as urolithiasis [2] are hard masses produced in the urinary tract and cause infection, pain, bleeding or obstruction. Multiple types of stones such as oxalate, uric acid, cysteine, or struvite stone can be produced in the urinary tract [3]. Infection stones include magnesium ammonium phosphate or struvite (which accounts to 10–15% of urinary stone), carbonate apatite and ammonium urate. Urease producing bacteria such as *Proteus* are responsible for production of urease enzyme which alkalinizes urine and produce these stones [4, 5]. Moreover, ammonia and oxalate stones damage the urothelial layer facilitating microbial invasion [6].

*E. coli*, a urease negative bacterium is the most common cause of UTI and dominant isolate in urinary stones and urine cultures. Its existence in stone indicates either urease negative bacteria have a role in stone formation or urease producing bacteria transiently infect and lost after stone formation [7]. Urease producing bacteria include *Proteus*,* Morganella*,* Pseudomonas, Providencia*, *Klebsiella* and *S. aureus* and are common causes of UTI [8]. *P. mirabilis* is the prominent urease producing species and cause of struvite calculi [9]. Bacteria incrusted in or attached on the surface of stone forming biofilm and cause recurrent and multidrug resistant (MDR) UTI [10]. The rate of UTI among urolithiasis patients ranges from 7 to 60% [11] with the coincidence of these comorbidities results in complications such as renal failure and death [12].

Urinary stone disease is one of the worldwide threats which has been increasing particularly in the last 15 years [13, 14]. Factors such as sex, previous history of UTI, condom use, vaginal infection, extremes of age, anatomical abnormalities like congenital urinary tract malformations, urinary obstruction, catheterization, resent sexual activity, comorbidities like urinary stone, diabetes and obesity [8, 15, 16], hospitalization, antibiotic use are risks factors to UTI [17]. Information about the burden of UTI among patients with urinary calculi is lacking in Ethiopia. This study is aimed to investigate bacterial uropathogens, antimicrobial resistance and associated factors among Urinary stone patients at the University of Gondar Comprehensive Specialized Hospital, Gondar Ethiopia.

## Materials and methods

### Study area, design, and period

A prospective cross-sectional study was conducted from January to April, 2019, at the University of Gondar Comprehensive Specialized Hospital. The hospital is located in Gondar town, Amhara regional state, Northwest Ethiopia, situated at 742 km from the capital city of Ethiopia, Addis Ababa. The hospital is a tertiary level teaching and referral hospital catering more than 500 beds for inpatients and rendering referral health services for over 5 million inhabitants in Northwest Ethiopia.

*Population* All patients who have visited the hospital during the study period and clinically suspected of urinary stone disease were the source population while patients with ultrasound confirmed urinary stone were study population. All the study participants were enrolled from the outpatient department.

*Exclusion criteria* Patients with history of antibiotic use in the last 2 weeks prior to diagnosis and during the data collection period were excluded or denied from the study because those who have been taking antibiotics may have culture negative due to their resent drug use. All patients who had urinary system complains, were confirmed to have urinary stone with ultrasound and not on antibiotics treatment in the last 2 weeks as well as during the study period and willing to be included were eligible to this study.

### Sample size and sampling methods

The sample size was calculated by a single population proportion formula taking 50% prevalence since previous study involving urinary stone patients was not available.

$${\text{n}} = \frac{{{\text{z}}_{\alpha /2}^{2} {\text{p}}\left( {1 - {\text{p}}} \right)}}{{{\text{d}}^{2} }}$$

$$= \frac{{1.96^{2} *0.5\left( {1 - 0.5} \right)}}{{\left( {0.05} \right)^{2} }} = 384$$

Using reduction formula as the total population based on the same period of the last year’s record, estimated population was 1350, the final sample size was

$${\text{n}}_{{\text{f}}} = \frac{{{\text{n}}*{\text{N}}}}{{{\text{n}} + {\text{N}}}}$$

$${\text{n}}_{{\text{f}}} = \frac{384*1350}{{384 + 1350}} = 299$$

where N is the total population, n_f_—the final sample size, Z_*α*/2_—the standard normal deviation, at 95% confidence level = 1.96, p—The prevalence = 0.5; 1 − p = 0.5 and d—The desired degree of accuracy = 0.05.

### Data collection and laboratory methods

A total of 300 study participants with urinary stone were enrolled in the study using convenient sampling technique. Sociodemographic and clinical features of study participants such as age, gender, residence, history of UTI and drug use, stone size and its position, presence of stone at multiple sites, condom use, catheterization and presence of comorbidites like history of diabetes, HIV were colleceted using structured questionaire. Clean catch mid-stream urine was collected in a sterile wide mouthed urine cup with prior adiquate instruction of the participant (Additional file 1).

### Culture and microbiological identification

Early morning mid-stream urine sample was collected and transported to medical bacteriology laboratory; inoculated in Cysteine lactose electrolyte deficient (CLED) agar (BIOMARK^R^ Laboratories, India) within two hours and incubated for 18–24 h at 35–37 °C. A 0.001 mL inoculating loop was used to inoculate the urine specimen. Colonies with significant bacteriuria (≥ 10^5^ CFU/mL) were subcultured on blood agar plate (BAP) and MacConkey agar for isolation of a single species and further characterization by hemolysis on BAP, lactose fermentation on MacConkey agar. Gram staining and biochemical tests such TSI, motility test, indole test, citrate test, urease test, Lysine decarboxylase, catalase, coagulase tests, novobiocin sensitivity, and Bile Esculin (BIOMARK^R^ Laboratories, India) hydrolysis test were used for species identification [18].

### Antimicrobial susceptibility testing

Antimicrobial susceptibility testing (AST) was performed using Kirby-Bauer disc diffusion method on Muller-Hinton agar (BIOMARK^R^ Laboratories, India). Selected antimicrobial agents such as Ampicillin (10 µg), Amoxicillin-clavulanate (20/10 µg), Ciprofloxacin (5 µg), Tobramycin (10 µg), Gentamicin (10 µg), Cefixime (5 µg), Cefoxitin (30 µg), Cefuroxime (30 µg), Nitrofurantoin (300 µg), Rifampin (5 µg), Tetracycline (30 µg), Penicillin 10 units, Norfloxacin (10 µg), Vancomycin (30 µg), Trimethoprim-sulphamethoxzole (1.25/23.75 µg) (Abtek Biologicals Ltd, United Kingdom) and Cefotaxime (30 µg), Ceftazidime (30 µg), Cefotaxime-Clavulanate (30/10 µg), Ceftazidime-Clavulanate (30/10 µg) (HiMedia Laboratories Pvt Ltd India) were used for antimicrobial susceptibility test [19]. Zones of inhibition were measured after 16–18 h of incubation at 37 °C and classified as susceptible or resistant. Isolates intermediate between susceptible and resistant were considered as resistant [20]. Isolates resistant to three or more classes of antibiotics were considered as MDR [21].

### Extended spectrum beta lactamase detection

First screening for ESBL production was done for Cefotaxime and Ceftazidime 30 µg each. Isolates with zone of inhibition ≤ 27 mm for cefotaxime and ≤ 22 mm for ceftazidime were phenotypically confirmed for ESBL production by using Ceftazidime-clavulanic acid (30 μg/10 μg), Cefotaxime-clavulanic acid (30 μg/10 μg) with their respective single disks using combined disk test. The suspension of screened isolates was prepared and inoculated on MHA. The target antibiotics were then placed at least 25 mm apart from each other followed by 16–18 h of incubation at 35 °C ± 2 °C and zone of inhibition was measured. At least 5 mm difference to either of the combined drugs and their respective single disks was considered as ESBL producing species [19].

*Quality control* The questionnaire was pretested for standardization and clearity. The culture media were checked for sterility and performance prior to inoculation by incubating 5% of the newly prepared media overnight. The reagents for Gram’s stain were also assured for their expiry date and staining. The control strains *E. coli* ATCC25922 and *S. aureus* ATCC25923 were used to control performance of media and antibiotic disks. Furthermore, *K. pneumoniae* ATCC 700603 and *E. coli* ATCC25922 were used as a positive and negative control for ESBL production respectively. The suspension was standardized against 0.5 McFarland standards [19].

*Data processing and analysis* Data were entered and analyzed using SPSS version 23 software. Discriptive statistics such as frequency and percentage were determined. The binary mogistic regression model was used. The assumption Hosmer–Lemeshow goodness of fit as well as cox and snell and Nagelkerke R squire valus were checked and the model was fit. Bivariate and multivariate logistic regression analysis were carried out with enter method to determine statistically significant association and odds ratio at 95% confidence interval was computed. Variables with a *P* value < 0.2 in bivariate logistic regression analysis were entered to multivariate logestic regression analysis [22, 23]. Variables with *P* value < 0.05 in multivariate logistic regression analysis were considered as statistically significant.

## Result

### Sociodemographic and clinical characteristics of patients with urinary stone

Of the 300 urinary stone patients enrolled in the present study, 153(51%) were male, 123(41%) were in age group 20–29 years, 261(87%) were urban dwellers and 134(44.7%) were patients with educational level of college and above. More than half, 176(58.7%) of participants had family monthly income 2000 Ethiopian birr and above (Table 1). Thirty-three (11%) of study participants had history of UTI, 25(8.3%) of them had stone size of 5 mm and above. Nine (3%) of the participants had history of urinary blockage and 166(55.3%) of them had history of antibiotic use while 17(5.7%) were hospitalized previously (Table 2).

**Table 1:** Sociodemographic characteristics of urinary stone patients at the University of Gondar Comprehensive Specialized Hospital, January to April 2019

Variables	Frequency (%)
Sex	
Male	153(51)
Female	147(49)
Age	
< 20 years	23(7.7)
20–29	123(41)
30–39	97(32.3)
40–49	32(10.7)
≥ 50	25(8.3)
Residence	
Urban	261(87)
Rural	39(13)
Educational level	
Cannot read and write	35(11.7)
Elementary	71(23.7)
High school and preparatory	60(20)
College and above	134(44.7)
Family monthly income (ETB)	
< 1000	54(18)
1001–2000	70(23.3)
> 2000	176(58.7)
Sexual activity	
Within 2 days	43(14.3)
Within 7 days	84(28)
Pregnancy (for females)	
Yes	6(2)
No	141(47)
Use of birth control mechanisms	
Condom	6(2)
No	141(46.3)
Body mass index	
< 18.5	40(13.3)
18.5–24.5	211(70.3)
> 24.5	49(16.3)

**Table 2:** Clinical characteristics of urinary stone patients at the University of Gondar Comprehensive Specialized Hospital, January to April 2019

Variable	Frequency (%)
History of UTI	
Yes	33(11)
No	267(89)
Urinary blockage	
Yes	9(3)
No	291(97)
History of catheterization	
Yes	4(1.3)
No	296(98.7)
Stone size	
< 5 mm	275(91.7)
≥ 5 mm	25(8.3)
Stones at multiple locations	
Yes	75(25)
No	225(75)
Location of stone	
Renal	282(94)
Renal and/or extra renal	18(6)
History of chronic disease	
Yes	21(7)
No	279(93)
History of drug use	
Yes	172(57.3)
No	128(42.7)
Drug type used	
Antibiotics	166(55.3)
Antibiotics, steroids and HAART	7(2.3)
Antibiotic use	
Within the past one month	70(23.3)
Within the past 3 months	41(13.7)
History of hospitalization	
Yes	17(5.7)
No	283(94.3)

Extra renal-(Kidney and ureter, Kidney and bladder, or Kidney and urethra)

### The prevalence of bacterial uropathogens among patients with urinary stone

**Fig. 1:**
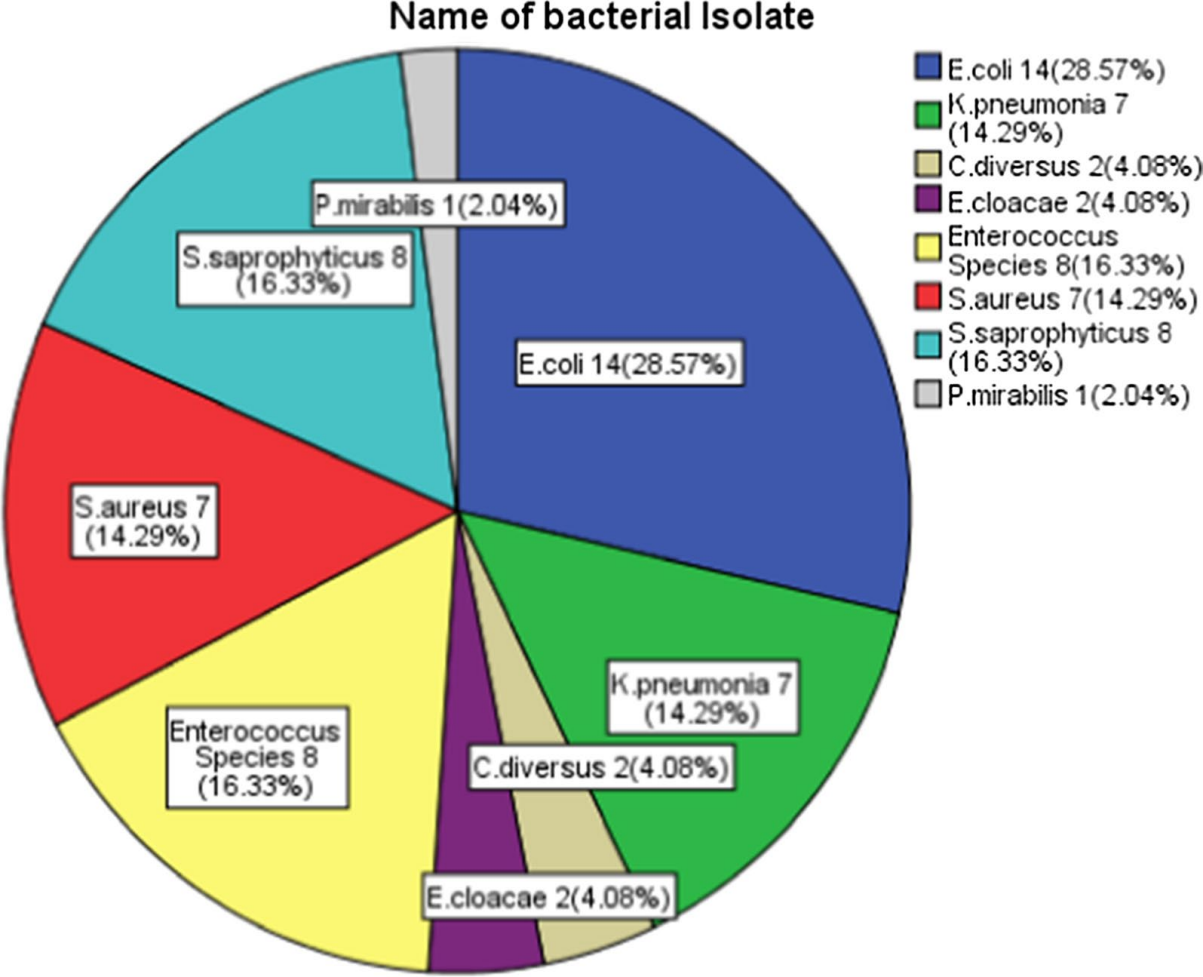
The proportion of bacterial uropathogens isolated from Urinary stone patients at the University of Gondar Comprehensive Specialized Hospital, January to April 2019

Forty-nine 49/300(16.3%) (95% CI 12–21%) of the urinary stone patients had UTI. The most common cause of UTI was *E. coli* 14/49(28.6%) followed by *S. saprophyticus* and *Enterococcus* species 8/49(16.3%) each, *K. pneumoniae* and *S. aureus* 7/49(14.3%) each (Fig. 1).

### Antimicrobial susceptibility of bacterial uropathogens among patients with urinary stone

Majority of Gram-negative isolates were resistant to ampicillin 25/26 (96.2%), amoxicillin-clavulanate 18/26 (69.2%) while 46/49 (94%) and 44/49(90%) of the Gram-negative isolates were susceptible to nitrofurantoin and ciprofloxacin respectively. All *S. saprophyticus* 8(100%) and 6/7 (85.7%) of *S. aureus* were susceptible to gentamycin. Two 2/8(25%) *S. saprophyticus* and 1/7 (14.3%) of *S. aureus* isolates were resistant to cefoxitin (surrogate marker for methicillin resistance), and one vancomycin resistant *Enterococcus* was isolated (Table 3).

**Table 3:** Antimicrobial Susceptibility Pattern of Bacterial Isolates from Urinary stone Patients at the UOGCSH, January to April 2019

Antibiotic used	Species of isolate with their antibiotic susceptibility							
	*E. coli* N (%)	*K. pneumoniae* N(%)	*C. diversus* N (%)	*E. cloacae* N (%)	*P. mirabilis* N (%)	*Enterococci* N (%)	*S. saprophyticus* N (%)	*S. aureus* N (%)
AMP								
S		1(14.3)				2(25)		
R	14(100)	6(85.7)	2(100)	2(100%)	1(100)	6(75)		
AMC								
S	2(14.3)	4(57.1)		1(50)	1(100)			
R	12(85.7)	3(42.9)	2(100)	1(50)				
CTX								
S	7(50)	6(85.7)	1(50)	2(100)	1(100)			
R	7(50)	1(14.3)	1(50)					
CAZ								
S	14(100)	6(85.7)	1(50)	2(100%)	1(100)			
R		1(14.3)	1(50)					
FOX								
S	13(92.9)	6(85.7)	2(100)	2(100%)	1(100)		5(62.5)	6(85.7)
R	1(7.1)	1(14.3)					3(37.3)	1(14.3)
CFM								
S	12(85.7)	4(57.1)	1(50)	2(100)	1(100)			
R	2(14.3)	3(42.9)	1(50)					
CRX								
S	13(92.9)	6(85.7)	1(50)	2(100)	1(100)			
R	1(7.1)	1(14.3)	1(50)					
CPR								
S	12(85.7)	6(85.7)	1(50)	2(100)	1(100)	8(100)	7(87.5)	7(100)
R	2(14.3)	1(14.3)	1(50)				1(12.5)	
GEN								
S	13(92.9)	7(100)	1(50)	2(100)	1(100)		8(100)	6(85.7)
R	1(7.1)		1(50)					1(14.3)
TOB								
S	10(71.4)	7(100)	1(50)	2(100)	1(100)			
R	4(28.6)		1(50)					
TET								
S	7(50)	4(57.1)		2(100)		3(37.5)	3(37.5)	5(71.4)
R	7(50)	3(42.9)	2(100)		1(100)	5(62.5)	5(62.5)	2(28.3)
SXT								
S	6(42.9)	4(57.1)		2(100%)	1(100)		6(75)	5(71.4)
R	8(57.1)	3(42.9)	2(100)				2(25)	2(28.6)
NIT								
S	14(100)	6(85.7)	2(100)	2(100%)	1(100)	8(100)	6(75)	7(100)
R		1(14.3)					2(25)	
NOR								
S	11(78.6)	6(85.7)	1(50)	2(100%)	1(100)	5(62.5)	7(87.5)	6(85.7)
R	3(21.4)	1(14.3)	1(50)			3(37.5)	1(12.5)	1(14.3)
PEN								
S						3(37.5)	2(25)	4(57.1)
R						5(62.5)	6(75)	3(42.9)
RIF								
S						6(75)		
R						2(25)		
VAN								
S						7(87.5)		
R						1(12.5)		

*AMP* Ampicillin, *AMC* Amoxicillin-clavulanate, *FOX* Cefoxitin, *CFM* Cefixime, *CRX* Cefuroxime, *CPR* Ciprofloxacin, *GEN* Gentamycin, *CAZ* Ceftazidime, *CTX* Cefotaxime, *RIF* Rifampin, *NOR* Norfloxacin, *NIT* Nitrofurantoin, *PEN* Penicillin, *RIF* Rifampin, *SXT* Trimethoprim-Sulfamethoxazole, *TET* Tetracycline, *TOB* Tobramycin, *VAN* Vancomycin

### MDR and ESBL producing uropathogenic isolates among patients with urinary stone

Of the total uropathogens, 16/49(32.7%) were MDR and 75% of which were Enterobacteriaceae. The most common MDR species were *E. coli* 7/49(14.3%), followed by *K. pneumoniae* 3/49(6.12%). Furthermore, from a total of 26 Enterobacteriaceae 9/26(34.6%) isolates were confirmed ESBL producing species. These confirmed ESBL producing isolates were *E. coli* 6/26 (23.1%) and *K. pneumoniae* 3/26(11.5%) (Table 4).

**Table 4:** The prevalence of MDR isolates from urinary stone patients at the University of Gondar Comprehensive Specialized Hospital, January to April 2019

Antibiotics	Species of bacteria					Total
	*E. coli*	*K. pneumoniae*	*C. diversus*	*Enterococci*	*S. saprophyticus*	
AMP, TET, NOR				2		2
AMP, TET, SXT		1				1
AMP, AMC, TOB, SXT	1					1
AMP, TET, TOB, SXT	1					1
AMP, AMC, TET, SXT			1			1
AMP, AMC, CTX, SXT	1					1
CXT, TET, SXT, PEN					1	1
CXT, TET, NOR, PEN					1	1
AMP, AMC, CTX, SXT, TET	1					1
AMP, AMC, CTX, CFM, SXT	1					1
AMP, FOX, CFM, CPR, NIT		1				1
AMP, AMC, CRX, TET, SXT		1				1
AMP, AMC, SXT, GEN, NOR, TET, TOB	1					1
AMP, AMC, CTX, CFM, GEN, TOB, TET, CPR, SXT, NOR			1			1
AMP, AMC, CTX, FOX, CFM, CRX, CPR, TOB, TET, NOR	1					1
Total	7	3	2	2	2	16
ESBL positive	6	3				9

*AMP* Ampicillin, *AMC* Amoxicillin-clavulanate, *CTX* cefotaxime, *FOX* Cefoxitin, *TET* Tetracycline, *CPR* Ciprofloxacin, *CTX* Cefotaxime, *PEN* Penicillin, *TOB* Tobramycin, *SXT* Trimethoprim-Sulfamethoxazole, *CRX* Cefuroxime, *GEN* Gentamycin, *NOR* Norfloxacin

### Factors associated with urinary tract infection among patients with urinary stone

Among the variables, sex, age, education, monthly income, history of UTI, blockage of urinary system, stone size, stone location in the urinary system, history of chronic disease and drug use, sexual activity, history of hospitalization, and body mass index had *P* value < 0.2 in bivariate analysis and were computed by multivariate logistic regression analysis to check statistically significant association by controlling possible confounders. The result of multivariate logistic regression showed that being female, history of UTI, drug use had a *P* value of < 0.05; which indicates statistically significant association with patients having urinary stone diseases (Table 5).

**Table 5:** Factors associated with UTI among urinary stone patients at the UOGCSH, January to April 2019

Variable	Significant bacteriuria		COR 95% CI	*P* value	AOR 95% CI	*P* value
	Yes (%)	No (%)				
Sex						
Male	13(26.5)	140(55.8)	1			
Female	36(73.5)	111(44.2)	3.5(1.77–6.90)	0.00	5.76(2.23–13.32)	0.00
Age						
< 20 years	3(6)	20(8)	1		1	
20–29	21(42.9)	102(40.6)	1.37(0.37–5.04)	0.63	2.00(0.43–9.26)	0.38
30–39	12(24.5)	85(33.9)	0.94(0.24–3.65)	0.93	1.05(0.25–6.57)	0.77
40–49	9(18.4)	23(9.16)	2.6(0.62–10.98)	0.19	2.04(0.38–14.92)	0.36
≥ 50 years	4(8.2)	21(8.4)	1.27(0.25–6.40)	0.77	1.25(0.16–12.16)	0.77
Residence						
Urban	42(85.7)	219(87.3)	1		1	
Rural	7(14.3)	32(12.7)	1.14(0.47–2.76)	0.77	1	
Education						
Cannot read and write	10(20.4)	25(10)	2.15(0.90–5.13)	0.08	1.80(0.54–4.5.95)	0.34
Elementary	13(28.3)	58(23.1)	1.21(0.56–2.58)	0.63	1.42(0.50–4.07)	0.51
High school	5(10.2)	55(21.9)	0.49(0.18–1.37)	0.17	0.31(0.09–1.02)	0.05
College and above	21(42.9)	113(45)	1		1	
Family monthly income (ETB)						
≤ 1000	16(32.7)	38(15.1)	2.95(1.413–6.15)	0.004	2.40(0.84–6.88)	0.104
1001–2000	11(22.4)	59(23.5)	1.31(.596–2.857)	0.50	1.60(0.61–4.20)	0.34
> 2000	22(44.9)	154(61.4)	1		1	
History of UTI						
Yes	15(30.6)	18(7.2)	5.71(2.63–12.38)	0.00	4.66(1.76–12.30)	0.002
No	34(69.4)	233(92.8)	1		1	
Urinary blockage						
Yes	3(6.1)	6(2.4)	2.66(0.64–11.03)	0.18	0.26(0.03–2.20)	0.22
No	46(93.9)	245(97.6)	1		1	
Stone size						
< 5 mm	43(87.8)	232(92.4)	1			
≥ 5	6(12.2)	19(7.6)	1.7(0.64–4.5)	0.28		
Stone location						
Renal	46(93.9)	236(94)	1			
Renal and/or extra renal	3(6)	15(6)	1.03(0.29–3.7)	0.97		
Chronic disease						
Yes	6(12.2)	15(6)	2.2(0.81–1.5.97)	0.12	1.17(0.24–5.65)	0.85
No	43(87.8)	236(94)	1		1	
Drug use						
Yes	36(73.5)	136(54.2)	1		1	
No	13(26.5)	115(45.8)	0.43(0.22–0.84)	0.014	0.33(0.14–0.77)	0.01
Hospitalization						
Yes	7(14.3)	10(4)	4.02(1.45–11.14)	0.008	3.85(0.92–16.07)	0.065
No	42(85.7)	241(96)	1		1	
Sexual activity						
Within 2 days	6(12.2)	37(14.7)	0.55(0.21–1.45)	0.23	0.61(0.20–1.92)	0.40
Within 7 days	11(22.4)	73(29.1)	0.51(0.24–1.10)	0.09	0.48(0.19–1.21)	0.12
Within a month	5(10.2)	49(19.5)	0.35(0.13–0.96)	0.041	0.30(0.086–1.02)	0.054
Never	27(55.1)	92(36.7)	1		1	
BMI						
18.5–24.5	32(65.3)	179(71.3)	1		1	
> 24.5	7(14.3)	42(16.7)	0.536(0.24–1.20)	0.13	1.24(0.41–3.79)	0.71
< 18.5	10(20.4)	30(12)	0.5(0.17–1.46)	0.21	2.89(0.98–8.50)	0.053

*AOR* adjusted odds ratio, *COR* crude odds ratio, *CI* confidence interval, *ETB* Ethiopian Birr

## Discussion

The overall prevalence of UTI among patients with urinary stone was 49/300 (16.3%) (95% CI 12–21%). This result is similar to reports by Gutierrez et al. 16.2% [24] and lower than a study done in china 22.0% [25], Iran 35.5% [26], Romania 25.8% [27] and India 45% [11] among urinary stone patients. However, this result was higher than 7.8% prevalence of UTI reported from Los Angeles, United States [28]. The variation might be because of the socioeconomic, geographical and population difference. The predominant species isolated in this study were *E. coli*, 14/49(28.6%) followed by *S. saprophyticus* and *Enterococcus* species 8/49 (16.3%) each. The result was comparable to other reports 33% *E.coli* and 18.5% *Enterococci* isolated from patients with urinary stone [29]. On the other hand the result was higher than 6.5% prevalence of *E.coli* reported by Gutierrez et al. [24].

The resistance to antibiotics such as ampicillin, amoxicillin-clavulanate and Trimethoprim-sulphamethoxzole in this study was 31/33(94%), 18/26(69.23%) and 17(41.5%) respectively which was slightly lower than a report in India ampicillin 96% and amoxicillin-clavulanate 87% [30]. The result of this study was higher than resistance to Ampicillin 70%, and Amoxicillin-clavulanic acid 30% reported in Romania [27]. The high rate of resistance might be due to easy access and self-medication, weak adherence of patients to prescribed antimicrobial agents, wider availability of empirical treatment in health care settings [12, 31, 32]. Most of the isolates identified were susceptible to nitrofurantoin 46/49(94%) and ciprofloxacin 44/49(90%). There were two methicillin resistant *S. saprophyticus* and one methicillin resistant *S. aureus* (MRSA) which was nearly similar to a case of methicillin resistant coagulase negative staphylococcus report in Romania [27].

From a total of 16 MDR isolates, *E. coli* 7/16(43.75%) and *K. pneumoniae* 3/16(18.75%) were the most common MDR species. The MDR isolates in this study were higher than 29.3% MDR *E. coli*, 6.8% *K. pneumoniae* but lower compared to 12.0% MDR *E. faecalis*. The difference could be due to sample size variation [33]. Furthermore, 9/26(34.6%) of the total 26 Enterobacteriaceae, were ESBL producing species. These ESBL producing species were *E. coli* 6/26(23%) and *K. pneumoniae* 3/26(11.5%) species. This result was lower than 51.8% of the overall prevalence of ESBL producing Enterobacteriaceae [12]. The prevalence of ESBL producing Enterobacteriaceae from the total *E.coli* isolates was higher than 8.7% result by Bianca et al. [31]. This might be due to variation in the management and use of antibiotics.

In the present study the prevalence of UTI was higher among female participants 36/49 (73.5%). This corresponds to a study done by Bianca et al. [31] 74.2% in females and 25.8% in males. The result was slightly higher than a 67% prevalence in females as compared to 33% in males [11]. The likelihood of acquiring UTI in females was 5.76 times higher compared to males (AOR 5.76, 95% CI 2.23–13.32). This could be due to the physio-anatomical variations between males and females including anal and genital proximity, short urethra as well as sexual activity in females which ease the access of intestinal flora to the urinary tract in females. On the other hand antimicrobial activity of the prostate secretions in males could contribute to the reduced infection rate among males than female participants [25, 27].

The finding of this study showed that 15/49(31%) of patients with significant bacteriuria had previous history of UTI. Having history of UTI increases the risk of UTI by 4.7 times (AOR 4.66, 95% CI 1.76–12.30) than those who had not. This might be due to the release of bacteria from the inside of stone or from its matrix protected surface resulting in persistence or recurrence of the UTI. Previous history of UTI is among the factors that predisposes to the occurrence of the infection [15]. The treatment might not have completely removed the etiologic agent of UTI in patients. Because the bacteria can be incrusted in the stone or extracellular matrix and escape from the treatment [34].

In addition, 36/49(73.5%) patients with significant bacteriuria had previous history of drug use and showed a statistically significant association with UTI. The risk of UTI among patients who had not history of drug use reduces by 67% compared to those who had history of drug use (AOR 0.33, 95% CI 0.14–0.77). This could be due to the fact that steroidal drugs are immunosuppressive and patients could be immunocompromised [30]. The increased antimicrobial resistance in drug users and the recurrence or persistence of drug resistant UTI might also contribute.

As a limitation only urine specimen was taken for culture and stone culture was not performed to which could help to know the agreement of stone and urine culture strain characterization was not performed due to lack of resources.

## Conclusion

The commonest bacterial species isolated from patients with urinary stone disease was *E. coli*. The most common MDR and ESBL producing isolates were *E. coli* and *K. pneumoniae.* Being female, previous history of UTI and drug use were the independent risk factors. Isolated uropathogens were most susceptible to nitrofurantoin, ciprofloxacin and gentamycin. However, most of the isolates were resistant to ampicillin, penicillin and trimethoprim-Sulfamethoxazole. Routine diagnosis of urinary stone patients for urinary tract infection should be promoted. Antimicrobial stewardship program should be implemented to reduce drug resistance.

## Supplementary Information

**Additional file 1**. English version of the questionnaire.

## Data Availability

The datasets used and/or analyzed during the current study are available from the corresponding author on reasonable request.

## Abbreviations

AST: Antimicrobial susceptibility test
ATCC: American Type Culture Collection
BAP: Blood agar plate
CLSI: Clinical Laboratory Standards Institute
CLED: Cysteine lactose electrolyte deficient agar
ESBL: Extended spectrum beta lactamase
HAART: Highly active antiretroviral therapy
MAC: MacConkey agar
MDR: Multidrug resistance
TSI: Triple sugar iron agar
USD: Urinary stone disease
UTI: Urinary tract infection

## References

[1] Schwaderer AL, Wolfe AJ (2017). The association between bacteria and urinary stones. Ann Transl Med.

[2] Kirkali Z, Rasooly R, Star RA, Rodgers GP (2015). Urinary stone disease: progress, status, and needs. Urology.

[3] Sakhaee K, Maalouf NM, Sinnott B (2012). Kidney stones 2012: pathogenesis, diagnosis, and management. J Clin Endocrinol Metab.

[4] Miano R, Germani S, Vespasiani G (2007). Stones and urinary tract infections. Urologia Int.

[5] Thomas B, Tolley D (2008). Concurrent urinary tract infection and stone disease: pathogenesis, diagnosis and management. Nat Rev Urol.

[6] Hassan JK (2011). Crystalluria types and incidence in Basra City; southern of Iraq. J Basrah Res (Sciences).

[7] Shojaeian A, Rostamian M, Noroozi J, Pakzad P (2016). The identification of chemical and bacterial composition and determination of FimH gene frequency of kidney stones of Iranian patients. J Res Med Sci.

[8] Flannigan R, Choy WH, Chew B, Lange D (2014). Renal struvite stones—pathogenesis, microbiology, and management strategies. Nat rev Urol.

[9] Norsworthy AN, Pearson MM (2017). From catheter to kidney stone: the uropathogenic lifestyle of *P. mirabilis*. Trends Microbiol.

[10] Romanova YM, Mulabaev N, Tolordava E, Seregin A, Seregin I, Alexeeva N (2015). Microbial communities on kidney stones. Mol Gen Microbiol Virol.

[11] Songra M, Damor M, Namdev RK, Patbamniya NK, Nawalakhe P, Jain R (2016). A study on positive stone culture and its association with rate of sepsis after urological procedures. Int Surg J.

[12] Chen D, Zhang Y, Huang J, Liang X, Zeng T, Lan C (2018). The analysis of microbial spectrum and antibiotic resistance of uropathogens isolated from patients with urinary stones. Int J Clin Pract.

[13] Raheem OA, Khandwala YS, Sur RL, Ghani KR, Denstedt JD (2017). Burden of urolithiasis: trends in prevalence, treatments, and costs. Eur Urol Focus.

[14] Scales CD, Smith AC, Hanley JM, Saigal CS (2012). Prevalence of kidney stones in the United States. Eur Urol.

[15] Foxman B (2014). Urinary tract infection syndromes: occurrence, recurrence, bacteriology, risk factors, and disease burden. Infect Dis Clin North Am.

[16] Tandogdu Z, Wagenlehner FM (2016). Global epidemiology of urinary tract infections. Curr Opin Infect Dis.

[17] Eshetie S, Unakal C, Gelaw A, Ayelign B, Endris M, Moges F (2015). Multidrug resistant and carbapenemase producing Enterobacteriaceae among patients with urinary tract infection at referral Hospital, Northwest Ethiopia. Antimicrob Resist Infect Control.

[18] Cheesbrough M (2006). District laboratory practice in tropical countries.

[19] CLSI. Performance Standards for Antimicrobial Susceptibility Testing. 28th ed. CLSI supplement M100. 2018.

[20] Tellevik MG, Blomberg B, Kommedal Ø, Maselle SY, Langeland N, Moyo SJ (2016). High prevalence of faecal carriage of ESBL-producing Enterobacteriaceae among children in Dar es Salaam, Tanzania. PLoS ONE.

[21] Goyal D, Dean N, Neill S, Jones P, Dascomb K, editors. Risk Factors for Community-Acquired Extended-Spectrum Beta-Lactamase–Producing Enterobacteriaceae Infections—A Retrospective Study of Symptomatic Urinary Tract Infections. In: Open forum Infect Dis; 2019; 6(12):ofz517. Oxford University Press. 10.1093/ofid/ofy357 PMC6366654 30775401

[22] Pasricha J, Koessler T, Harbarth S, Schrenzel J, Camus V, Cohen G (2013). Carriage of extended-spectrum beta-lactamase-producing enterobacteriacae among internal medicine patients in Switzerland. Antimicrob Resist Infect Control.

[23] Kin-On K, Emily C, Chung P-H, Arthur T, Wan-In W, Chendi Z (2020). Prevalence and associated factors for carriage of Enterobacteriaceae producing ESBLs or carbapenemase and Methicillin-resistant Staphylococcus aureus in Hong Kong Community. J Infect.

[24] Gutierrez J, Smith A, Geavlete P, Shah H, Kural AR, de Sio M (2013). Urinary tract infections and post-operative fever in percutaneous nephrolithotomy. World J Urol.

[25] Yongzhi L, Shi Y, Jia L, Yili L, Xingwang Z, Xue GJ (2018). Risk factors for urinary tract infection in patients with urolithiasis primary report of a single center cohort. BMC Urol.

[26] Shafi H, Shahandeh Z, Heidari B, Sedigiani F, Ramaji AA, Pasha YRY (2013). Bacteriological study and structural composition of staghorn stones removed by the anatrophic nephrolithotomic procedure. Saudi J Kidney Dis Transpl.

[27] Maier A, Man A, Chibelean C, Cighir T, Nemes-Nagy E, Maier I (2015). Bacteriological evaluation of the non-struvite nephrolithiasis and its association with urinary tract infections. Rev Rom Med Lab.

[28] Abrahamian FM, Krishnadasan A, Mower WR, Moran GJ, Talan DA (2013). Association of pyuria and clinical characteristics with the presence of urinary tract infection among patients with acute nephrolithiasis. Ann Emerg Med.

[29] Tavichakorntrakool R, Prasongwattana V, Sungkeeree S, Saisud P, Sribenjalux P, Pimratana C (2012). Extensive characterizations of bacteria isolated from catheterized urine and stone matrices in patients with nephrolithiasis. Nephrol Dial Transplant.

[30] Bora DI, Lyngdoh DW, Khyriem DB (2015). Bacteriological profile of rena. Int J Curr Res.

[31] Bianca T, Adrian M, Emil M, Adrian T (2013). Microbiological study of urinary calculi in patients with urinary infections. Acta Medica Transilv.

[32] Bell BG, Schellevis F, Stobberingh E, Goossens H, Pringle M (2014). A systematic review and meta-analysis of the effects of antibiotic consumption on antibiotic resistance. BMC Infect Dis.

[33] Wang S, Zhang Y, Zhang X, Li J (2020). An evaluation of multidrug-resistant (MDR) bacteria in patients with urinary stone disease: data from a high-volume stone management center. World J Urol.

[34] Hedelin H (2002). Uropathogens and urinary tract concretion formation and catheter encrustations. Int J Antimicrob Agents.

