# Supplementary material for: Multiple drug resistance bacterial isolates and associated factors among urinary stone patients at the University of Gondar Comprehensive Specialized Hospital, Northwest Ethiopia
Source: BMC Urol. 2021 Feb 23;21:27. doi: 10.1186/s12894-021-00794-8 (PMC7901194; doi:10.1186/s12894-021-00794-8)
Supplement: Supplementary file 1 — Additional file 1. English version of the questionnaire. [file 12894_2021_794_MOESM1_ESM.docx]

**Annex II: A. Questionnaire English version**

| 1. **Socio demographic characteristics** | | | | |
| --- | --- | --- | --- | --- |
| **S. No** | Participant ID | | | ___________________ |
| 1 | Gender of the participant | | | 1. Male 2. Female |
| 2 | Age of the participant | | | _______________ |
| 3 | Residence | | | 1. Urban  2. Rural  |
| 4 | Pregnancy | | | 1. Yes  2. No  |
| 5 | Use of birth control mechanisms | | | 1. Condom  2. Diaphragm/ vaginal ring  3. spermicide  |
| 6 | Educational level | | | 1. Unable to read and write  2. Able to read and write 3. Elementary school  4. High school  5. College and above  |
| 7 | Family monthly income | | | 1. Less than 500 Birr  2. 501-1000 Birr  3. 1001-1500 Birr  4. 1501-2000 Birr  5. More than 2000 Birr  |
| 1. **Clinical information** | | | | |
| 1 | History of urinary tract infection | | | 1. Yes  2. No  |
| 2 | History of catheter use | | | 1. Yes 2. No |
| 3 | Blockage of the urinary system | | | 1. Yes  2. No |
| 1. **Risk factors of urinary tract infection among urinary stone patients** | | | | |
| 1 | | stone size | 1. Less than 5 mm  2. B 5-10 mm  3. 10-20 mm  4. > 20 mm  | |
| 2 | | Location of stone in the urinary system | 1. Kidney  2. B Ureter  3. bladder  4. Urethra  | |
| 3 | | Presence of stone at multiple locations | 1. Kidney  2. B urether  3. bladder  4. Urethra  | |
| 4 | | History of chronic diseases | 1. Yes  2. B No  | |
| 5 | | If yes which disease | 1. diabetes  2. C. Hypertension  3. Heart disease  4. HIV infection 5. Others mention | |
| 6 | | Prior drug use | 1. Yes  2. No  | |
| 7 | | Type of drug used | 1. Antibiotics  2. steroidal drugs  3. HAART  4. Cancer chemotherapy  | |
| 8 | | Antibiotic use with in the past | 1. 1 month  2. 3monthes  3. 6 monthes  4. 1year | |
| 9 | | Hospitalization | 1. Yes  2. No  | |
| 10 | | If yes to question 9 time of hospitalization within the past | 1. 1moth  2. 3monthes  3. 6monthes  4. 1year  | |
| 11 | | Sexual activity | 1. Within 2 days  2. within 7 days  3. A week ago  4. Never  | |
| 12 | | Body mass index | 1. Less than 18.5  2. 18.5-24.5  3. Above 24.5 | |
